# When the Urine Turns Purple: Not Always a Urinary Tract Infection

**DOI:** 10.7759/cureus.97200

**Published:** 2025-11-19

**Authors:** Elisa Veigas, João Lança Pereira, Nidia Oliveira, Andreia Lopes, Ana Filipa Viegas

**Affiliations:** 1 Internal Medicine, Unidade Local de Saúde de Viseu Dão-Lafões, Viseu, PRT

**Keywords:** asymptomatic bacteriuria, bed rest, purple urine bag, tryptophan metabolism, urinary catheter, urinary tract infection

## Abstract

Purple urine bag syndrome (PUBS) is an uncommon but distinctive phenomenon characterized by violet discoloration of the urinary collection system. It occurs mainly in elderly, debilitated, and chronically catheterized patients, and although alarming, it is usually benign. We report the case of an 89-year-old woman with end-stage renal disease on hemodialysis and long-term catheterization who developed purple discoloration of the urine bag during hospitalization for influenza A. She remained asymptomatic, and the discoloration resolved after catheter replacement and optimization of bowel care without antibiotic therapy. PUBS results from bacterial metabolism of tryptophan into indigo and indirubin pigments that adhere to catheter surfaces, a process favored by constipation and chronic colonization. Recognition of this mechanism is crucial to distinguish PUBS from urinary tract infection and to prevent unnecessary antibiotic use. This case underscores the importance of clinical awareness and appropriate catheter management to ensure safe, evidence-based care in chronically catheterized patients.

## Introduction

The study and interpretation of urine dates back to ancient medicine. Hippocrates, often regarded as the first uroscopist, recognized the diagnostic value of urine in assessing disease and humoral imbalance. Centuries later, Theophilus Protospatharius wrote "De Urinis", the first treatise devoted entirely to urine, followed by the 15th-century "Fasciculus Medicinae" of Johannes de Ketham, which introduced the famous urine wheel illustrating different urine colours and their meanings [1,2].

Normal urine owes its yellow color primarily to urochrome, identified in the 19th century, with minor contributions from urobilin and uroerythrin. Variations in these pigments, hydration status, pH, or exposure to air explain most color changes. Endogenous pigments include hemoglobin, myoglobin, bilirubin, uric acid, and homogentisic acid, whereas exogenous causes are linked to certain drugs, dyes, foods, and toxins such as propofol, indigo, beetroot, and phenol [3].

Described for the first time in 1978, purple urine bag syndrome (PUBS) is a rare and visually distinctive finding marked by a violet discoloration of the urine collection system [4].

PUBS results from bacterial metabolism of tryptophan into indigo and indirubin pigments that stain the urinary collection system. It mainly affects elderly, immobilized, and chronically catheterized patients, particularly women [4-8]. Although often associated with bacteriuria, PUBS does not necessarily indicate an active urinary tract infection. Clinical evaluation remains essential to differentiate benign colonization from true infection, avoid unnecessary antibiotic therapy, and guide appropriate catheter care [7].

We present a case of PUBS in an elderly woman, emphasizing its characteristic appearance and benign course.

This article was previously presented as a poster at the 31º Congresso Nacional de Medicina Interna on May 22-25, 2025, Portugal.

## Case presentation

An 89-year-old woman, fully dependent and residing in a nursing home, was admitted with influenza A and suspected bacterial superinfection. She was treated with oseltamivir 30 mg after each dialysis session for five days and ceftriaxone 2 g intravenously once daily for seven days for a respiratory infection. Her past medical history included end-stage chronic kidney disease on hemodialysis for over five years due to obstructive uropathy with cystopathy, chronic indwelling urinary catheterization for more than 10 years (with residual urine output despite dialysis), anemia of chronic disease, dyslipidemia, and chronic constipation. Regular medications included erythropoietin beta (6,000 IU weekly), folic acid, atorvastatin, lactulose, furosemide due to preserved residual diuresis, and calcitriol supplementation.

**Figure 1:**
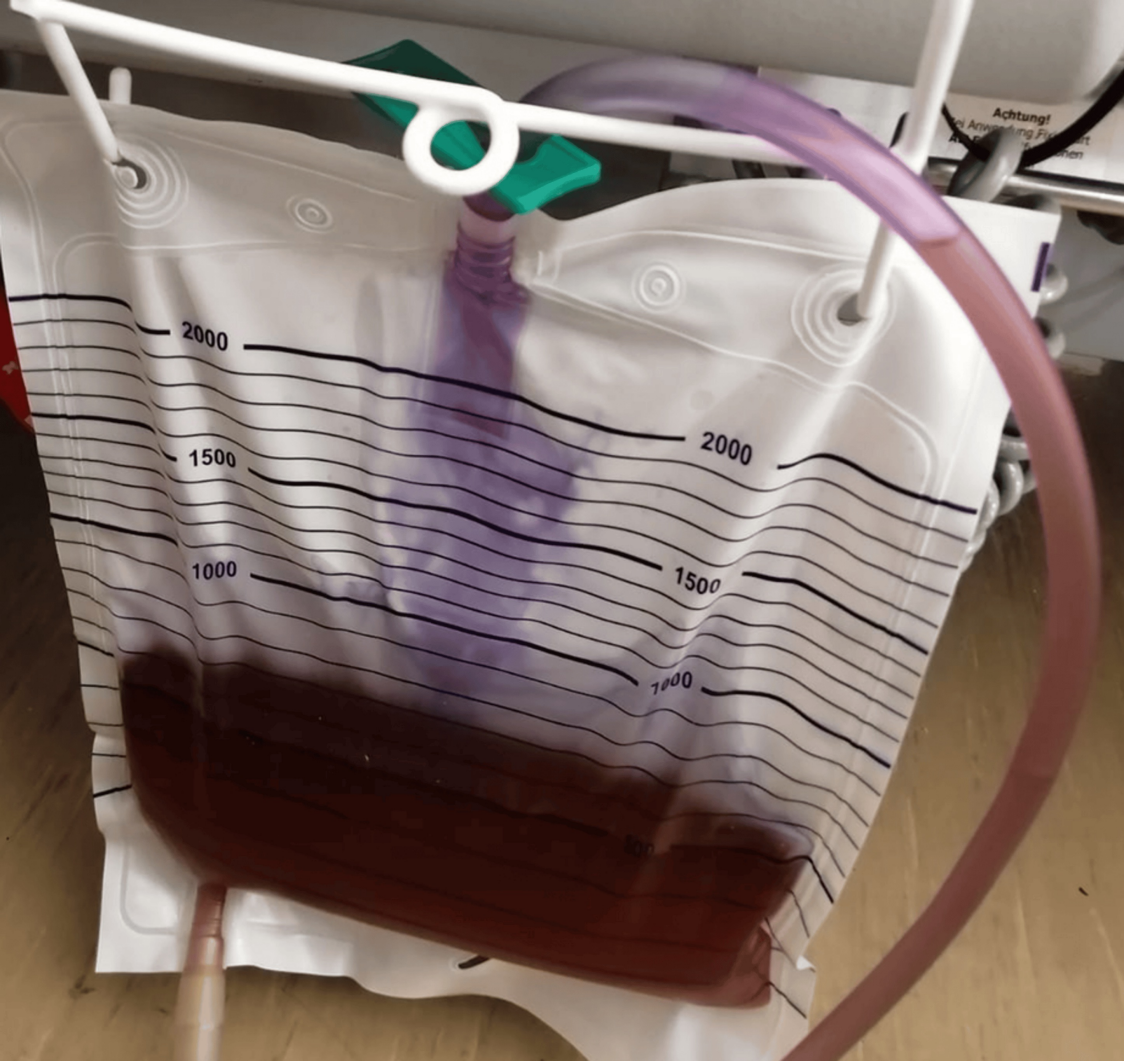
Characteristic appearance of PUBS. Violet discoloration of the urinary drainage bag due to pigment deposition typical of PUBS. PUBS: purple urine bag syndrome

On admission, laboratory findings revealed mild leukopenia and thrombocytopenia with elevated procalcitonin levels (Table 1). During the initial days of hospitalization, violet discoloration of the urine drainage bag was observed (Figure 1).

**Table 1:** Laboratory parameters on admission and at discharge. Initial results showing mild leukopenia, thrombocytopenia, and elevated procalcitonin levels.

Laboratory parameter	On admission	At discharge	Reference range
Leucocytes (x10^9^/L)	3.55	4.45	4.50-11.50
Platelets (x10^9^/L)	112	169	150-450
Hemoglobin (g/dL)	9.8	10.2	12-16
Serum Ionogram	Normal	Normal	-
Procalcitonin (ng/mL)	1.36	0.35	< 0.50
C-reactive protein (mg/dL)	4.85	2.57	< 0.50
Blood cultures	Different growth of gram-positive cocci		
Multiplex panel by PCR detection	Positive Flu A (negative for Flu B and SARS-CoV-2)		

On admission, laboratory findings revealed mild leukopenia and thrombocytopenia with elevated procalcitonin levels. During the initial days of hospitalization, violet discoloration of the urine drainage bag was observed (Figure 1). The patient remained afebrile, hemodynamically stable, and without urinary symptoms. Inflammatory markers progressively improved. Given the absence of fever, urinary complaints, or clinical evidence of infection, urine culture was not obtained. In this case, management consisted solely of catheter and drainage system replacement, with optimization of laxative therapy. No antibiotics were initiated for this finding. The discoloration resolved spontaneously and did not recur during the remainder of her seven-day hospital stay or after discharge.

## Discussion

The reported incidence of PUBS is approximately 9.8% among chronically catheterized institutionalized patients [5]. In hospitalized populations, prevalence ranges from 8.3% to 42.1% [6], emphasizing the importance of recognizing this condition among healthcare professionals caring for catheterized patients.

PUBS arises from bacterial metabolism of tryptophan. In the gut, tryptophan is degraded to indole, which is absorbed and converted in the liver to indoxyl sulfate (indican). This colorless metabolite is excreted in urine, where bacteria with sulfatase and phosphatase activity, most commonly *Providencia stuartii* and *Providencia rettgeri*, but also *Proteus mirabilis*, *Klebsiella pneumoniae*, *Pseudomonas aeruginosa*, *Escherichia coli*, *Morganella morganii*, *Citrobacter *species, *Enterococcus*, and Group B *Streptococcus*, hydrolyze it to indoxyl. In an alkaline environment, indoxyl is oxidized to the pigments indigo (blue) and indirubin (red), which combine and adhere to the synthetic material of the catheter and drainage bag, producing the characteristic purple discoloration [6-8].

Multiple factors predispose to PUBS. Chronic constipation, bowel stasis, and prolonged urinary catheterization promote bacterial overgrowth and increased production of indoxyl sulfate [3,6]. Elderly, bedridden, and institutionalized patients are particularly vulnerable, and the condition affects women more frequently (≈70%) due to anatomical factors (shorter urethra and its proximity to the anus) that favor colonization by gram-negative bacilli [6,9].

Other predisposing factors include dehydration, recurrent urinary infection, dementia, and end-stage renal disease [6]. In chronic kidney disease, accumulation of protein-bound toxins such as indoxyl sulfate is common, and clearance is further reduced in hemodialysis patients. Alkaline urine predominates in most cases (≈91%), though PUBS may occur even in acidic urine [6].

Additional contributors include the use of polyvinyl chloride (PVC) drainage systems, high urinary bacterial load [8], and, more rarely, ileal conduits or *Clostridioides difficile *infection [10].

Despite its dramatic appearance, PUBS is typically harmless and resolves once the catheter is replaced and the contributing factors are corrected. For those unfamiliar with the condition, the sudden discoloration often triggers unnecessary concern among staff and relatives, but awareness of its benign nature prevents overtreatment [8].

Management of PUBS focuses on treatment of urinary tract infection when present, on maintaining proper catheter care and removing predisposing factors [7,8,11].

Although PUBS is typically benign and self-limiting, it should not lead to diagnostic complacency. Each episode must be evaluated in the context of the individual patient’s clinical condition, as urinary tract infections can coexist and may require treatment. Careful assessment of symptoms, signs, and laboratory parameters remains essential to differentiate asymptomatic bacteriuria from true infection, particularly in frail or immunocompromised patients [11].

In this case, PUBS developed after the patient was already receiving systemic ceftriaxone for a respiratory infection, suggesting that the discoloration occurred independently of antibiotic therapy. This temporal relationship reinforces the concept that, in this patient, PUBS resolution was primarily achieved through catheter replacement and correction of predisposing factors (correction of constipation and optimization of hydration) rather than antimicrobial treatment.

No urine culture was obtained because the patient remained afebrile, asymptomatic, and without laboratory evidence of infection. The decision was based on clinical judgment rather than omission, as PUBS developed in the context of chronic catheterization and stable inflammatory markers. This approach is consistent with current international guidelines, including those from the Infectious Diseases Society of America (IDSA, 2019) and the Association for Professionals in Infection Control and Epidemiology (APIC, 2025), all of which recommend against obtaining urine cultures or treating asymptomatic bacteriuria in catheterized patients without clinical evidence of infection [12,13].

Supportive measures include ensuring a closed drainage system, minimizing catheter duration, and performing daily hygiene of the meatus and perineal skin with clean technique. The drainage bag should be replaced every five to seven days or earlier if discoloration, odor, or leakage occur [14]. Preventive guidelines emphasize maintaining a closed drainage circuit, keeping the collection bag below bladder level, emptying it when two-thirds full, ensuring unobstructed urine flow, and reassessing the indication for catheterization every 48-72 hours [15].

Rather than signifying an active infection, PUBS usually reflects chronic colonization associated with prolonged catheterization and poor drainage hygiene. The inappropriate use of antibiotics in such cases has contributed to the emergence of multidrug-resistant organisms, *Clostridioides difficile* infection, and other drug-related adverse effects, highlighting the importance of antibiotic stewardship [16].

Although the prognosis is generally favorable, rare cases have progressed to severe complications, such as septic shock and Fournier’s gangrene, emphasizing the need for proper catheter care and regular clinical reassessment [6,17].

The present case fits the typical profile of patients affected by PUBS, an elderly, bedridden woman with chronic catheterization and constipation, but no evidence of active urinary tract infection. The condition resolved after catheter replacement and correction of predisposing factors, without the need for antibiotics, supporting the benign and self-limiting nature of this syndrome.

## Conclusions

PUBS is a vivid reminder that not every unusual clinical sign represents a serious condition. In many cases, it reflects the intersection of aging, chronic illness, and long-term catheterization rather than true infection. In this case, PUBS developed in an elderly woman with chronic catheterization and constipation, resolving after catheter replacement and bowel care. Recognizing PUBS prevents diagnostic confusion, avoids unnecessary investigations, and promotes antibiotic stewardship. Beyond its visual peculiarity, the syndrome underscores the importance of routine observation of catheterized patients, meticulous catheter care, and individualized clinical judgment. Each episode of PUBS should prompt clinicians to reassess the need for catheterization; evaluate symptoms, signs, and laboratory findings to determine the necessity of antibiotic therapy; and address modifiable risk factors, thereby improving outcomes through simple, evidence-based, and individualized care.
